# Specificity of malaria rapid diagnostic tests is affected by *Trypanosoma brucei gambiense* sleeping sickness

**DOI:** 10.1186/1475-2875-11-S1-P64

**Published:** 2012-10-15

**Authors:** Philippe Gillet, Jan Jacobs, Dieudonné Mumba Ngoyi, Albert Lukuka, Viktor Kande, Benjamin Atua, Johan Van Griensven, Jean-Jacques Muyembe, Philippe Büscher, Veerle Leion

**Affiliations:** 1 Department of Clinical Sciences, Institute of Tropical Medicine, Antwerp, Belgium; 2 lnstitut National de Recherche Biomédical, Kinshasa, Democratic Republic of the Congo; 3 Programme National de Lutte contre la Trypanosomiase Humaine Africaine (PNLTHA), Kinshasa, Democratic Republic of the Congo; 4 Programme National de Lutte contre le Paludisme (PNLP), Kinshasa, Democratic Republic of the Congo; 5 Department of Biomedical Sciences, Institute of Tropical Medicine, Antwerp, Belgium

## Background

In endemic settings, diagnosis of malaria increasingly relies on the use of rapid diagnostic tests (RDTs) instead of microscopic examinations. False positivity of such RDTs is poorly documented, although it may be particularly relevant in infections for which the differential diagnosis includes malaria, such as sleeping sickness, a fatal but treatable disease caused by *Trypanosoma brucei* parasite subspecies. We therefore examined the effect of *Trypanosoma brucei gambiense* sleeping sickness on the specificity of malaria RDTs.

## Materials and methods

Blood samples of 117 sleeping sickness patients and 117 matched non-sleeping sickness controls were prospectively collected in the Democratic Republic of the Congo. Reference malaria diagnosis was based on microscopy corrected by a four primer real-time PCR. Ten commonly used rapid diagnostic tests for malaria were evaluated including three two-band tests and seven three-band tests, based on the detection of Pf-HRP-2, Pf-pLDH and/or pan-pLDH antigens of *Plasmodium.*

## Results

Specificity of RDTs for diagnosis of malaria in controls was between 97.5 and 100% and was between 11.3 and 98.8% in sleeping sickness patients. For seven out of 10 RDTs, specificity was significantly lower in sleeping sickness patients compared to controls. Decreased specificity of malaria RDTs in sleeping sickness was mainly caused by false positivity of the pan-pLDH test lines, but also occurred frequently for the HRP-2 test lines.The *Pf*-pLDH test lines were not affected.

## Conclusions

Specificity of some malaria RDTs in sleeping sickness is surprisingly low, and constitutes a considerable risk for misdiagnosis or delayed diagnosis of sleeping sickness.

